# The Vi Capsular Polysaccharide of Salmonella Typhi Promotes Macrophage Phagocytosis by Binding the Human C-Type Lectin DC-SIGN

**DOI:** 10.1128/mbio.02733-22

**Published:** 2022-10-26

**Authors:** Lillian F. Zhang, Bernd Lepenies, Sayuri Nakamae, Briana M. Young, Renato L. Santos, Manuela Raffatellu, Brian A. Cobb, Hirotaka Hiyoshi, Andreas J. Bäumler

**Affiliations:** a Department of Medical Microbiology and Immunology, University of California at Davis, Davis, California, USA; b Institute for Immunology & Research Center for Emerging Infections and Zoonoses, University of Veterinary Medicine Hannover, Hanover, Germany; c Department of Immune Regulation, Shionogi Global Infectious Diseases Division, Institute of Tropical Medicine, Nagasaki University, Nagasaki, Japan; d Department of Pediatrics, School of Medicine, University of California San Diego, La Jolla, California, USA; e Chiba University-UC San Diego Center for Mucosal Immunology, Allergy, and Vaccines (CU-UCSD cMAV), La Jolla, California, USA; f Department of Pathology, School of Medicine, Case Western Reserve University, Cleveland, Ohio, USA; g Department of Bacteriology, Institute of Tropical Medicine, Nagasaki University, Nagasaki, Japan; University of Utah

**Keywords:** C-type lectin receptors, *Salmonella*, capsule, typhoid fever

## Abstract

Capsular polysaccharides are common virulence factors of extracellular, but not intracellular bacterial pathogens, due to the antiphagocytic properties of these surface structures. It is therefore paradoxical that Salmonella enterica subspecies *enterica* serovar Typhi, an intracellular pathogen, synthesizes a virulence-associated (Vi) capsule, which exhibits antiphagocytic properties. Here, we show that the Vi capsular polysaccharide has different functions when S. Typhi interacts with distinct subsets of host phagocytes. The Vi capsular polysaccharide allowed S. Typhi to selectively evade phagocytosis by human neutrophils while promoting human macrophage phagocytosis. A screen of C-type lectin receptors identified human DC-SIGN as the receptor involved in macrophage binding and phagocytosis of capsulated S. Typhi. Consistent with the anti-inflammatory activity of DC-SIGN, purified Vi capsular polysaccharide reduced inflammatory responses in macrophages. These data suggest that binding of the human C-type lectin receptor DC-SIGN by the Vi capsular polysaccharide contributes to the pathogenesis of typhoid fever.

## INTRODUCTION

In an immunologically naive host, bacterial entry into tissue triggers nonspecific binding of B-1 cell-derived natural immunoglobulin (Ig) M to a broad spectrum of polysaccharides on the bacterial surface, which leads to complement activation through the classical pathway (1). The resulting opsonization promotes bacterial uptake by professional phagocytes, an innate immune function aimed at killing the intruding microbes to restore tissue sterility. Bacterial pathogens can overcome these innate host defenses using virulence factors that allow them to either (i) evade opsonization and phagocytosis, which is the strategy used by extracellular pathogens, or (ii) evade killing during phagocytosis and take residence within the phagocyte, which is the approach taken by intracellular pathogens.

Polysaccharide capsules are a class of virulence factors that protect bacteria from opsonization with B-1 cell-derived natural IgM (2, 3), thereby preventing engulfment by phagocytic host cells such as neutrophils and macrophages (4). Due to their antiphagocytic properties, capsules are common virulence factors of extracellular pathogens that seek to evade uptake by professional phagocytes, including Staphylococcus aureus (5), Streptococcus pneumoniae (6), Streptococcus pyogenes (7), Neisseria meningitidis (8), Haemophilus influenzae (9), and Klebsiella pneumoniae (10). Although it makes sense that the antiphagocytic properties of capsules promote the extracellular lifestyles of the aforementioned pathogens, this strategy seems counterproductive for intracellular pathogens which seek to take residence within professional phagocytes to promote their persistence in tissue. It is therefore paradoxical that one important intracellular pathogen, Salmonella enterica subspecies *enterica* serovar Typhi, possesses an antiphagocytic capsule.

The strictly human-adapted S. Typhi is the causative agent of typhoid fever, a severe disseminated infection characterized by persistence of the pathogen in small granulomas, termed typhoid nodules, which are accumulations of mononuclear phagocytes and lymphocytes (11, 12). S. Typhi synthesizes a virulence-associated (Vi) capsular polysaccharide, also known as the Vi-antigen (13), which is encoded by the *viaB* locus (14) on Salmonella pathogenicity island 7 (SPI7) (15). The *viaB* locus contains genes for the regulation (*tviA*), biosynthesis (*tviBCDE*), and export (*vexABCDE*) of the Vi capsular polysaccharide (16, 17), which is a linear homopolymer of α-1,4 2-deoxy-2-*N*-acetylgalactosamine uronic acid variably *O*-acetylated at the C3 position (18). Vi capsular polysaccharide chains are anchored in the outer membrane of S. Typhi through a terminal *N*-acetylhexosamine residue modified with two beta-hydroxyl acyl chains (19). The Vi capsular polysaccharide prevents the binding of B-1 cell-derived natural IgM to the surface of S. Typhi (2, 3), thereby averting complement deposition through the classical pathway (2, 20, 21), complement-dependent neutrophil chemotaxis (22), phagocytosis (20), and the neutrophil respiratory burst (2, 23, 24). However, despite synthesizing an antiphagocytic capsule, S. Typhi resides intracellularly in tissues within phagocytes of the macrophage/monocyte lineage.

Here, we explore the duality of the S. Typhi Vi capsular polysaccharide by testing the hypothesis that the Vi capsular antigen allows S. Typhi to selectively evade phagocytosis by neutrophils, while promoting macrophage phagocytosis, by binding scavenger receptors synthesized by the latter cell type.

## RESULTS

### The Vi capsule prevents phagocytosis by neutrophils, but not macrophages.

**FIG 1:**
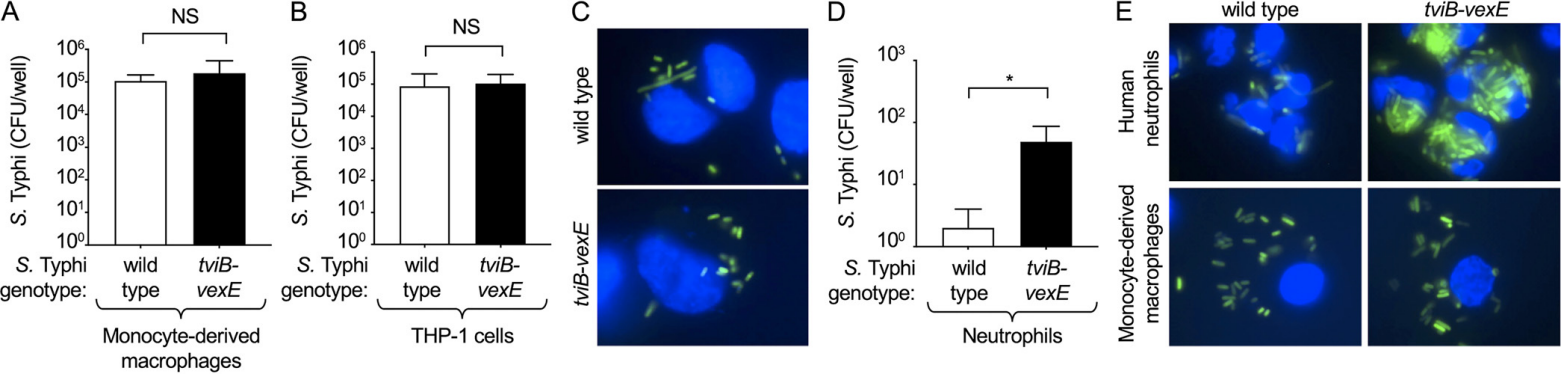
The virulence-associated (Vi) capsular antigen inhibits phagocytosis by neutrophils, but not macrophages. Human monocyte-derived macrophages (A and E), macrophage-like THP-1 cells (B and C) or human neutrophils (D and E) were infected with Vi capsulated Salmonella enterica subspecies *enterica* serovar Typhi (S. Typhi; wild type) or a noncapsulated S. Typhi (Δ*tviB-vexE* mutant). (A, B, and D) Recovery of CFU from a gentamicin protection assay 1 h after infection. Bars indicate geometric means ± standard error from 4 (A and B) or 3 (D) biological repeats. (C and E) Phagocytosis of GFP (green fluorescent protein)-labeled Vi capsulated S. Typhi (wild type) or a GFP-labeled noncapsulated S. Typhi *tviB-vexE* mutant (green fluorescence) by macrophage-like THP-1 cells (C), human neutrophils (E, top panels) or human monocyte-derived macrophages (E, bottom panels) was visualized 1 h after infection by fluorescence microscopy. Cells were counterstained with Hoechst nuclear stain (blue fluorescence). NS (non-significant), *P* > 0.05; *, *P* < 0.05.

We first investigated phagocytosis of S. Typhi by neutrophils and macrophages using a gentamicin protection assay. Primary human monocytes were isolated from the peripheral blood of healthy donors and differentiated into macrophages. These primary human monocyte-derived macrophages were then infected with either wild-type S. Typhi strain ATCC 700931 (Ty2) or an isogenic mutant carrying a precise deletion of the Vi capsule biosynthesis and export genes (S. Typhi Δ*tviB-vexE* mutant) (25). The cells were treated with gentamicin to kill extracellular bacteria, and intracellular bacteria were enumerated. There was no significant difference in recovery of capsulated (wild-type) or noncapsulated S. Typhi (Δ*tviB-vexE* mutant) from human monocyte-derived macrophages, suggesting that the Vi capsular polysaccharide does not impact association with macrophages (Fig. 1A). Similar results were obtained with human monocyte-like (THP-1) cells which had been differentiated into macrophages using phorbol 12-myristate-13-acetate (PMA) treatment (Fig. 1B). S. Typhi strains were transformed with a plasmid (pDW5) encoding green fluorescent protein (GFP). Microscopic analysis showed that green fluorescent S. Typhi (wild type or Δ*tviB-vexE* mutant) which was associated with THP-1 cells (Fig. 1C) could only be stained with antibodies against the O12 antigen of lipopolysaccharide (LPS) when THP-1 cells had been permeabilized with saponin (Fig. S1), suggesting that green fluorescent bacteria had an intracellular location. Notably, extracellular bacteria (i.e., bacteria stained with anti-O12 antiserum in the absence of saponin) no longer showed green fluorescence, suggesting that killing by gentamicin resulted in loss of the GFP label.

10.1128/mbio.02733-22.1 FIG S1 Human macrophage-like THP-1 cells were infected with Salmonella enterica subspecies *enterica* serovar Typhi (S. Typhi) expressing GFP (green fluorescent protein, left panels) for one hour using a gentamicin protection assay. Staining with anti-O12 serum (Alexa Fluor 594, red fluorescence, middle panels) was performed in the absence of saponin (bottom panels) or after permeabilization with saponin (top panels). Right panels show an overlay of GFP, O12, Hoechst nuclear stain (blue fluorescence), and bright field. The outline of THP-1 cells visualized by bright field microscopy (right panels) is indicated by white dashed lines (panels in the middle and right). Red dashed circles indicate extracellular bacteria which stain with anti-O12 (middle top panel) but are no longer GFP-positive (left top panel). Download FIG S1, PDF file, 0.2 MB. Copyright © 2022 Zhang et al. 2022 Zhang et al. https://creativecommons.org/licenses/by/4.0/ This content is distributed under the terms of the Creative Commons Attribution 4.0 International license.

Next, primary human neutrophils were isolated from the peripheral blood of healthy donors and infected with either capsulated S. Typhi (wild type) or an isogenic noncapsulated mutant (S. Typhi Δ*tviB-vexE* mutant). In contrast to the results obtained with macrophages, there were significantly more noncapsulated than capsulated S. Typhi recovered from primary human neutrophils, which was consistent with the idea that expression of the Vi capsular polysaccharide plays a role during pathogen interaction with neutrophils (Fig. 1D).

Reduced bacterial recovery from gentamicin protection assays can result from reduced bacterial uptake or enhanced bacterial killing. To distinguish between these possibilities, we performed microscopic analysis using S. Typhi strains carrying a plasmid (pDW5) encoding GFP. Microscopic analysis suggested that the reduced recovery of capsulated S. Typhi from primary human neutrophils compared to noncapsulated S. Typhi was due to reduced uptake of capsulated bacteria (Fig. 1E). In contrast, microscopy suggested that human monocyte-derived macrophages internalized capsulated and noncapsulated S. Typhi at similar levels.

### Vi capsular polysaccharide is bound by the C-type lectin DC-SIGN.

**FIG 2:**
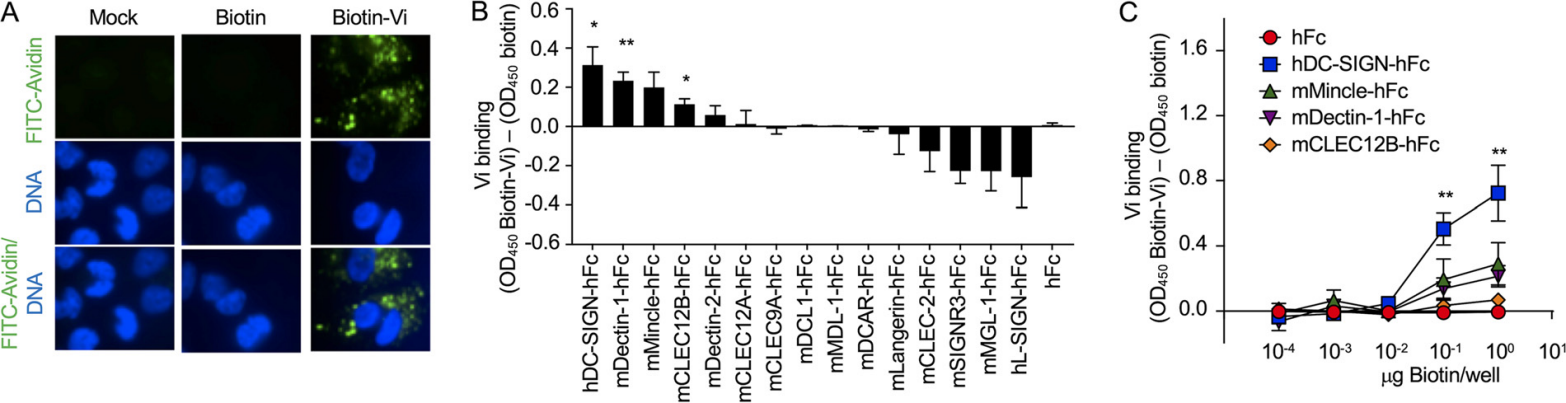
Purified Vi capsular polysaccharide binds the C-type lectin receptor (CLR) DC-SIGN. (A) Binding of biotinylated Vi capsular polysaccharide (Biotin-Vi) or biotin to macrophage-like THP-1 cells was visualized using FITC (fluorescein isothiocyanate)-labeled avidin (FITC-Avidin, green fluorescence). Macrophage-like THP-1 cells were counterstained with Hoechst nuclear stain (blue fluorescence). (B) Streptavidin-coated microplates were coated with Biotin-Vi or biotin, and binding to the indicated murine (m) or human (h) CLR proteins fused to the Fc portion of human immunoglobulin G_1_ (IgG_1_) (hFc) was assessed by enzyme-linked immunosorbent assay (ELISA). (C) Streptavidin-coated microplates were coated with increasing concentrations of biotin or biotin-Vi, and binding to the indicated CLR fusion proteins was measured by ELISA. (B and C) Graphs show signal (optical density at 450 nm [OD_450_]) generated by binding to biotin-Vi-coated wells which was higher than the background levels generated by binding to biotin-coated wells. Bars indicate geometric means ± standard error from 6 biological repeats. *, *P* < 0.05; **, *P* < 0.01.

We reasoned that the Vi capsular polysaccharide might not reduce phagocytosis by macrophages because it binds to a scavenger receptor displayed on the surface of this cell type. To test this possibility, we purified Vi capsular polysaccharide and labeled it with biotin. Consistent with our hypothesis, we were able to label THP-1-derived macrophages with biotinylated Vi capsular polysaccharide, but not with biotin (Fig. 2A). We next sought to identify a receptor that could recognize and bind to the Vi capsular polysaccharide. Because the Vi capsular polysaccharide is composed of repeating carbohydrate units, we predicted that a carbohydrate-binding protein, such a C-type lectin receptor (CLR), might be responsible for binding the capsular polysaccharide. CLRs contain one or more carbohydrate-recognition domains that can recognize a wide variety of glycans (26–28). We used a library of 15 CLR proteins fused to the Fc portion of human IgG_1_ (hFc) (29, 30), along with purified hFc as a negative control, to identify potential CLRs which bind biotinylated Vi capsular polysaccharide using an enzyme-linked immunosorbent assay (ELISA) screen. The identities of each of the CLR-hFc fusion proteins are provided in Fig. 2B. The CLR-hFc fusion proteins which bound biotinylated Vi capsular polysaccharide significantly better than biotin included human DC-SIGN (dendritic cell-specific intercellular adhesion molecule-3-grabbing non-integrin), murine Dectin-1, and murine CLEC12B (C-type lectin domain family 12 member B) (Fig. 2B). Binding was also observed when increasing concentrations of biotin or biotinylated Vi capsular polysaccharide were added to wells coated with CLR-hFc fusion proteins (Fig. 2C).

**FIG 3:**
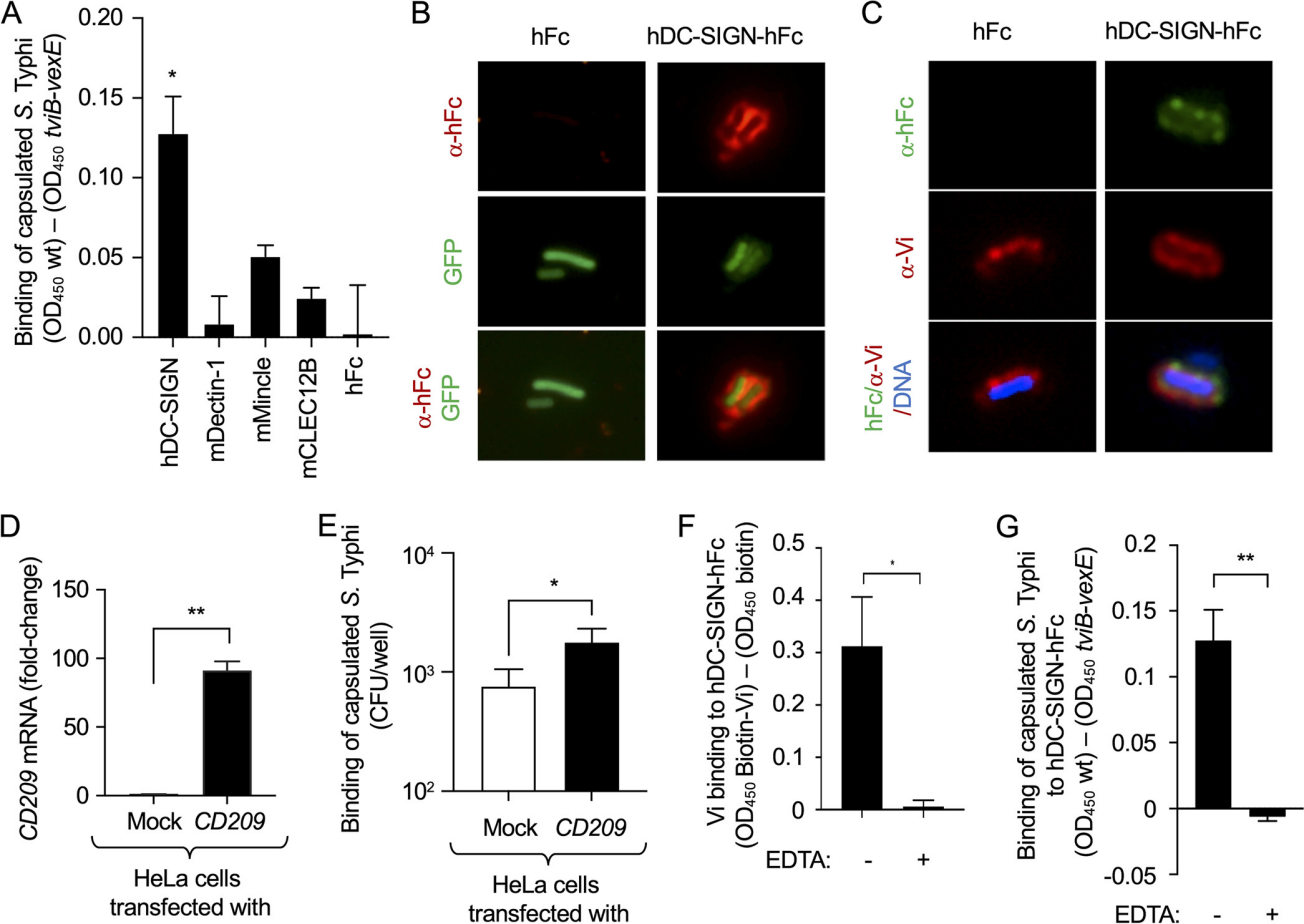
Vi capsulated S. Typhi binds human DC-SIGN. (A and G) Wells were coated with formalin-killed capsulated S. Typhi (wild type [wt]) or formalin-killed noncapsulated S. Typhi (*tviB-vexE*), and binding of the indicated murine (m) or human (h) CLR proteins fused to the Fc portion of human IgG_1_ (hFc) was assessed by ELISA. Graphs show signal (OD_450_) generated by binding to wt-coated wells which was higher than the background levels generated by binding to *tviB-vexE*-coated wells. (A) Bars indicate geometric means ± standard error from 4 biological repeats. (B) Binding of hFc or hDC-SIGN-hFc fusion protein (red fluorescence) to the surface of GFP-labeled capsulated S. Typhi (green fluorescence) was visualized by fluorescence microscopy. (C) Binding of hFc or hDC-SIGN-hFc fusion protein (green fluorescence) to the surface of S. Typhi (Hoechst DNA stain, blue fluorescence) synthesizing the Vi capsular antigen, detected by staining with anti-Vi antibodies (red fluorescence), was visualized by fluorescence microscopy. (D and E) Human cervical carcinoma epithelial (HeLa) cells were either mock treated (mock) or transfected with a plasmid encoding *CD209*. (D) Transcription levels of *CD209* were measured using quantitative real-time PCR. (E) The number of capsulated S. Typhi (CFU) adhering to HeLa cells was determined. (D and E) Bars indicate geometric means ± standard error from 3 biological repeats. (F) Streptavidin-coated microplates were coated with Biotin-Vi or biotin, and binding to hDC-SIGN-hFc was assessed by ELISA in the presence (+) or absence (–) of EDTA. Graphs show signal (OD_450_) generated by binding to biotin-Vi-coated wells that was higher than background levels generated by binding to biotin-coated wells. Bars indicate geometric means ± standard error from 5 biological repeats. (G) Binding to hDC-SIGN-hFc was assessed by ELISA in the presence (+) or absence (–) of EDTA. Bars indicate geometric means ± standard error from 3 biological repeats. *, *P* < 0.05; **, *P* < 0.01.

Previous work suggests that human DC-SIGN binds *N-*acetylglucosamine residues in the LPS core of Escherichia coli and S. Typhimurium (31). To determine whether the CLR-hFc fusion proteins bind to Vi capsular polysaccharide chains when they are displayed on intact bacterial cells, we evaluated binding of CLR-hFc fusion proteins to wells coated with formalin-killed capsulated (wild-type) or noncapsulated (Δ*tviB-vexE* mutant) S. Typhi strains. Wells coated with formalin-killed capsulated S. Typhi bound human DC-SIGN-hFc fusion protein significantly better than wells coated with formalin-killed noncapsulated S. Typhi (Fig. 3A), although the latter synthesizes an intact LPS core. Binding of DC-SIGN-hFc to the surface of wild-type S. Typhi was visualized by microscopy (Fig. 3B) and found to colocalize with staining for the Vi capsular polysaccharide (Fig. 3C). Next, we determined whether DC-SIGN synthesis increased binding of live S. Typhi to host cells. Transfection of cervical carcinoma epithelial (HeLa) cells with a plasmid encoding *CD209*, the gene encoding DC-SIGN, resulted in a marked increase in *CD209* transcription levels detected by quantitative real-time PCR (Fig. 3D) and significantly increased binding of live S. Typhi detected in an adhesion assay (Fig. 3E).

CLRs derive their name from the calcium dependence of their carbohydrate-binding activity (C-type stands for calcium-dependent type). Calcium ions are required for ligand binding and the structural integrity of the carbohydrate-recognition domains of CLRs (32). To determine whether binding of the Vi capsular polysaccharide to DC-SIGN exhibited the canonical calcium dependence, binding of DC-SIGN-hFc to wells coated with biotinylated Vi-capsular polysaccharide or formalin-killed capsulated S. Typhi was assessed in the presence and absence of EDTA, a Ca^2+^ chelating agent. Consistent with canonical C-type lectin binding, EDTA treatment abrogated binding of DC-SIGN-hFc fusion protein to biotinylated Vi-capsular polysaccharide (Fig. 3F) and formalin-killed capsulated S. Typhi (Fig. 3G).

Collectively, our results suggested that the human scavenger receptor DC-SIGN mediates calcium-dependent binding of the Vi capsular polysaccharide synthesized by S. Typhi.

### DC-SIGN is a macrophage receptor contributing to phagocytosis of S. Typhi.

**FIG 4:**
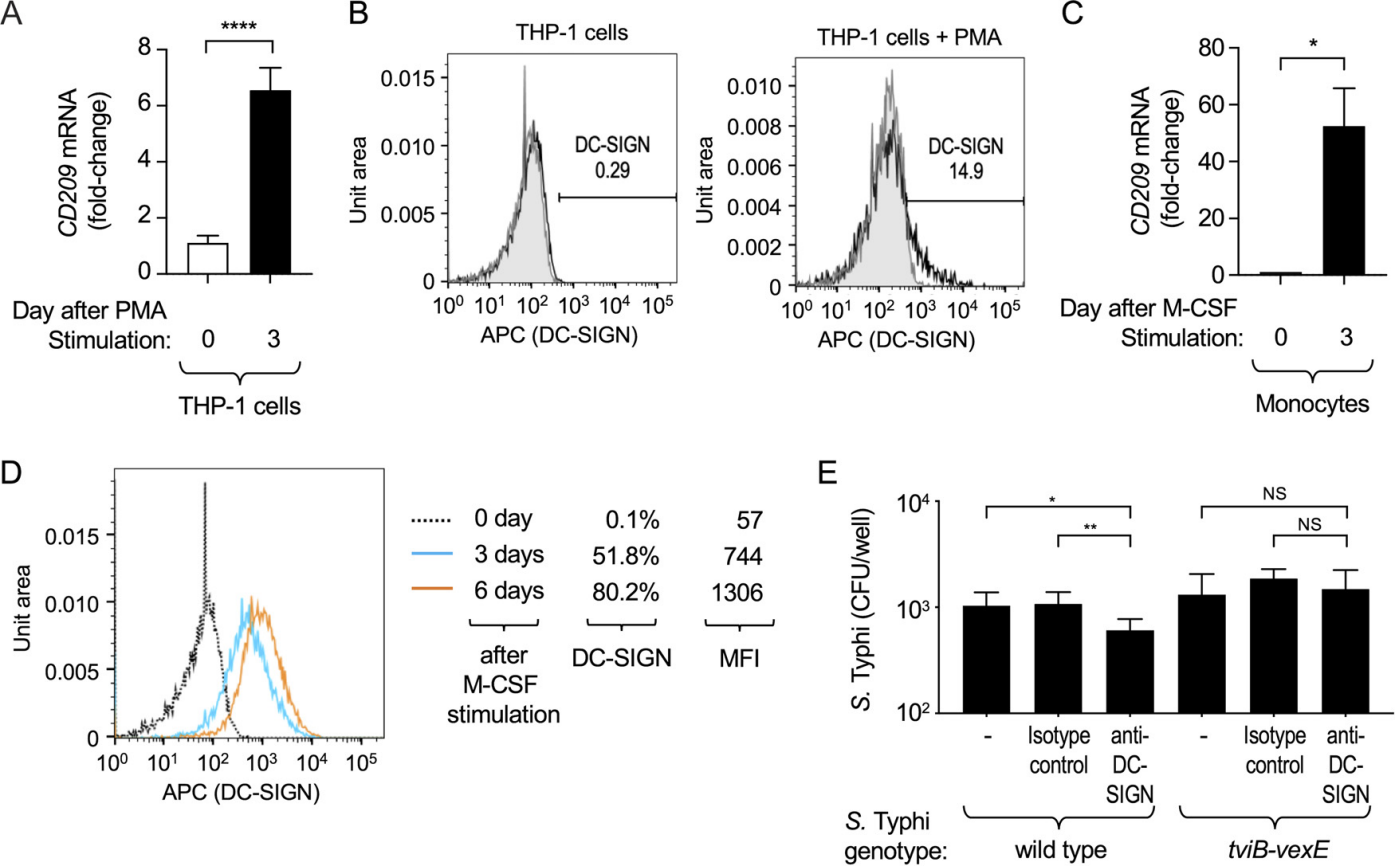
DC-SIGN present on macrophages contributes to phagocytosis of Vi capsulated S. Typhi. (A and C) Transcription levels of *CD209* were determined by quantitative real-time PCR in THP-1 cells (A) and human monocytes (C). The graphs show fold changes in *CD209* transcription levels prior to stimulation with phorbol 12-myristate-13-acetate (PMA) (A) or macrophage colony-stimulating factor (M-CSF) (C) compared to transcription levels detected 3 days after differentiation into macrophage-like THP-1 cells (A) or monocyte-derived macrophages (C). Bars indicate geometric means ± standard error from 7 (A) or 3 (C) biological repeats. (B and D) Synthesis of DC-SIGN on the surfaces of THP-1 cells (B) and human monocytes (D) was detected by flow cytometry using APC-labeled anti-DC-SIGN antibody. (B) DC-SIGN levels before stimulation with PMA (left panel) and after 3 days of stimulation with PMA (right panel). (D) DC-SIGN levels before stimulation with macrophage colony-stimulating factor (day 0 M-CSF, dotted line) and after 3 (blue line) or 6 days (orange line) of stimulation with M-CSF. MFI, mean fluorescence intensity. (E) Macrophage-like THP-1 cells were infected with Vi capsulated S. Typhi (wild type) or a noncapsulated S. Typhi *tviB-vexE* mutant in the presence of an anti-DC-SIGN blocking antibody or an isotype control antibody. Recovery of CFU from a gentamicin protection assay 1 h after infection. Bars indicate geometric means ± standard error from 6 biological repeats. NS, *P* > 0.05; *, *P* < 0.05; **, *P* < 0.01; ****, *P* < 0.001.

DC-SIGN has been reported to be present on the surface of human macrophages but not those of monocytes or neutrophils (33). Consistent with this report, using PMA to differentiate monocyte-like THP-1 cells into macrophage-like cells was accompanied by a 6-fold increase in transcription levels of *CD209*, the gene encoding DC-SIGN (Fig. 4A), and increased synthesis of DC-SIGN at the cell surface (Fig. 4B).

Macrophage colony-stimulating factor (M-CSF)-mediated differentiation of primary human monocytes into monocyte-derived macrophages was accompanied by an approximately 50-fold increase in *CD209* mRNA levels (Fig. 4C) and increased synthesis of DC-SIGN at the cell surface (Fig. 4D). These data confirmed that *CD209* expression and DC-SIGN synthesis were induced during the differentiation of monocytes into the macrophages used in this study.

To determine whether DC-SIGN contributes to phagocytosis of capsulated S. Typhi (wild type) we assessed uptake by macrophage-like THP-1 cells using a gentamicin protection assay in the presence of anti-DC-SIGN blocking antibodies or isotype control antibodies. Treatment of macrophage-like THP-1 cells with the anti-DC-SIGN blocking antibody significantly decreased the uptake of capsulated S. Typhi (wild type) compared to cells treated with the isotype control antibody (Fig. 4E). In contrast, treatment of macrophage-like THP-1 cells with the DC-SIGN blocking antibody did not reduce phagocytosis of the noncapsulated S. Typhi (Δ*tviB-vexE* mutant). These results supported the idea that DC-SIGN-mediated binding of the Vi capsular polysaccharide enhances phagocytosis of S. Typhi by macrophages.

### Binding of the Vi capsular polysaccharide to macrophages dampens proinflammatory responses.

**FIG 5:**
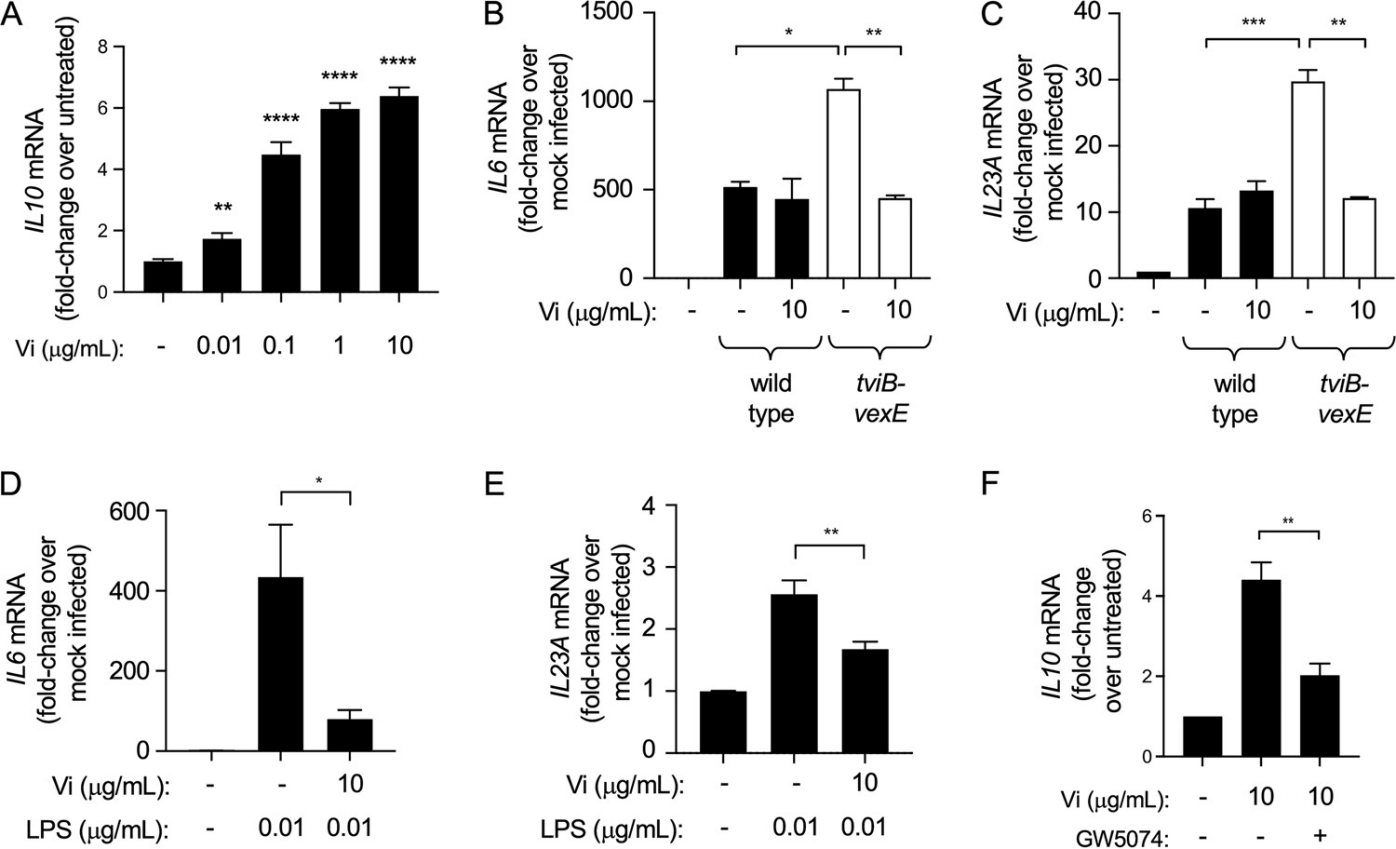
Binding of Vi capsular polysaccharide to macrophages dampens proinflammatory cytokine responses. (A) Macrophage-like THP-1 cells were stimulated with the indicated concentrations of purified Vi capsular polysaccharide and *IL-10* mRNA levels were determined by quantitative real-time PCR. Bars indicate geometric means ± standard error from 5 biological repeats. (B and C) Macrophage-like THP-1 cells were infected with the capsulated S. Typhi wild type or a noncapsulated S. Typhi (Δ*tviB-vexE* mutant) in the presence or absence of purified Vi capsular polysaccharide. Transcription levels of *IL-6* (B) and *IL-23A* (C) were determined by quantitative real-time PCR. Bars indicate geometric means ± standard error from 3 biological repeats. (D and E) Macrophage-like THP-1 cells were stimulated with lipopolysaccharide (LPS) in the presence or absence of purified Vi capsular polysaccharide. Transcription levels of *IL-6* (D) and *IL-23A* (E) were determined by quantitative real-time PCR. Bars indicate geometric means ± standard error from 5 biological repeats. (F) Macrophage-like THP-1 cells were stimulated with purified Vi capsular polysaccharide in the presence or absence of the RAF-1 inhibitor GW5074. Transcription levels of *IL-10* were determined by quantitative real-time PCR. Bars indicate geometric means ± standard error from 5 biological repeats. *, *P* < 0.05; **, *P* < 0.01; ***, *P* < 0.005; ****, *P* < 0.001.

Previous work suggests that several bacterial and viral pathogens, including Mycobacterium tuberculosis, M. leprae, Candida albicans, measles virus, and HIV-1, bind human DC-SIGN to activate the serine and threonine kinase RAF-1 (rapidly accelerated fibrosarcoma-1); the signaling pathway leads to an increase in interleukin (IL)-10 synthesis and a reduction in proinflammatory cytokine responses (34). Therefore, we wanted to investigate whether binding of DC-SIGN by the Vi capsular polysaccharide would also result in diminishing proinflammatory cytokine responses. Consistent with the finding that DC-SIGN binding induces IL-10 synthesis (34), treatment of THP-1-derived macrophages with purified Vi capsular polysaccharide induced expression of *IL-10* in a concentration-dependent manner (Fig. 5A). In agreement with the idea that the Vi capsule reduces inflammatory responses elicited by S. Typhi, infection of THP-1-derived macrophages with noncapsulated S. Typhi (Δ*tviB-vexE* mutant) increased expression of the proinflammatory cytokine genes *IL-6* and *IL-23A* compared to that in cells infected with capsulated S. Typhi (wild type) (Fig. 5B and C). Furthermore, addition of purified Vi capsular polysaccharide to THP-1-derived macrophages infected with noncapsulated S. Typhi reduced *IL-6* and *IL-23A* transcription levels to those observed with capsulated S. Typhi. Similarly, *IL-6* and *IL-23A* expression induced by stimulating THP-1-derived macrophages with purified lipopolysaccharide was blunted when cells were treated with purified Vi capsular polysaccharide (Fig. 5D and E).

To determine whether the Vi capsular polysaccharide increases *IL-10* expression through a RAF-1-dependent pathway, THP-1-derived macrophages were treated with a RAF-1 inhibitor (GW5074). *IL-10* expression induced by stimulation with purified Vi capsular polysaccharide was diminished by treatment with GW5074, suggesting that the Vi capsular polysaccharide induced *IL-10* expression through the DC-SIGN/RAF-1 pathway (Fig. 5F).

## DISCUSSION

**FIG 6:**
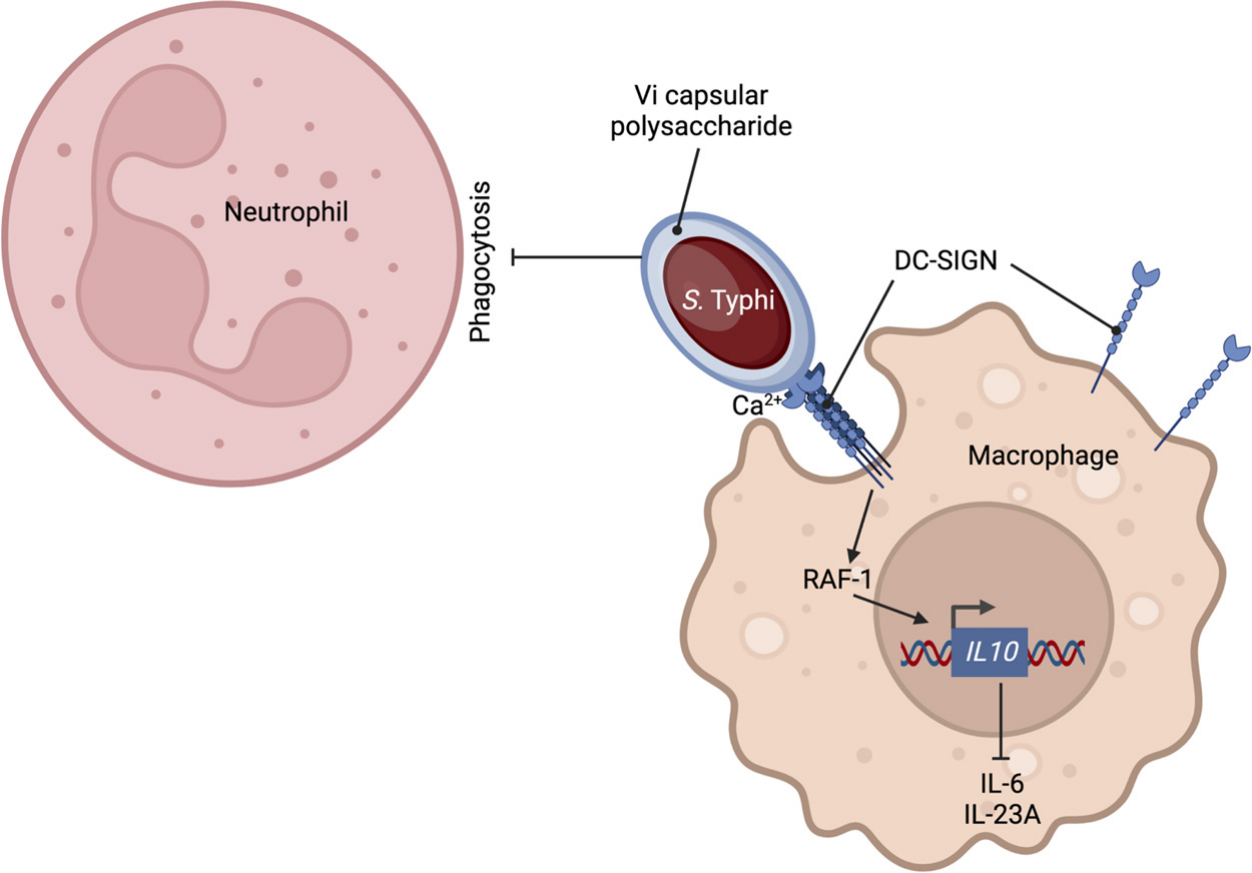
Model for the role of the Vi capsular polysaccharide in directing S. Typhi toward phagocytosis by macrophages.

Capsular polysaccharides are virulence factors used by extracellular bacterial pathogens to evade uptake and killing by professional phagocytes. It is therefore puzzling that an important intracellular bacterial pathogen, S. Typhi, synthesizes a capsular polysaccharide, the Vi antigen (13, 35). Here, we show that the Vi capsular antigen has a dual function: on the one hand, it exhibits antiphagocytic properties toward neutrophils, but on the other hand, it promotes macrophage phagocytosis. However, DC-SIGN-mediated phagocytosis of capsulated S. Typhi and phagocytosis of noncapsulated S. Typhi, which is complement-dependent (20), resulted in internalization of similar numbers of bacteria by macrophages. Thus, binding of the Vi capsular polysaccharide to DC-SIGN compensates for blocking complement-dependent phagocytosis. By inhibiting neutrophil phagocytosis while at the same time maintaining a similar level of uptake by macrophages, the Vi antigen directs S. Typhi toward taking residence within its preferred host phagocyte, at least during *in vitro* culture (Fig. 6). Unfortunately, one notable limitation of working with the strictly human-adapted S. Typhi is a lack of suitable animal models, which prevents analysis of how synthesis of the Vi capsular polysaccharide might influence the association of the pathogen with different host cell types *in vivo* (36). An *in vivo* analysis is further complicated by our finding that the Vi capsular polysaccharide binds to DC-SIGN, a CLR that is restricted to higher primates and has no true orthologue in mice (37). A paralogous gene in mice, *CD209s*, encodes a CLR that binds the LPS core of S. Typhimurium (38). DC-SIGN is present on the surface of both human macrophages and dendritic cells (33), where it has been linked to phagocytosis of fungal and bacterial pathogens (39, 40). Our data support the idea that DC-SIGN is a phagocytic receptor involved in the uptake of capsulated S. Typhi by human macrophages.

Binding of bacterial, fungal, or viral pathogens to DC-SIGN results in a dampening of proinflammatory cytokine responses (34). Consistent with these reports, we show that binding of purified Vi capsular polysaccharide by macrophages reduced expression of genes encoding proinflammatory cytokines. In addition to binding DC-SIGN, the capsule covers the bacterial surface so that the underlying LPS molecules can no longer be bound by natural IgM, thereby preventing complement activation (2). In turn, inhibition of complement activation dampens inflammatory responses elicited by LPS because complement component 3 fragment a (C3a) binds to its receptor on macrophages to increase expression of the gene encoding caspase-11, an intracellular LPS sensor (41). Thus, the anti-inflammatory activity of the Vi capsular polysaccharide is likely multifactorial, involving a DC-SIGN-mediated dampening of cytokine responses and an inhibition of complement activation. Furthermore, the activator of Vi capsular polysaccharide gene expression, TviA, serves as a repressor of genes encoding flagella and the invasion-associated type III secretion system (T3SS-1) (25). TviA-mediated repression of flagella and T3SS-1 synthesis helps limit inflammation by preventing Toll-like receptor (TLR)-5 activation (42), reducing RIP2 (receptor-interacting serine/threonine-protein kinase 2) activation (43), dampening inflammasome activation (44), and impairing proliferation of FliC-specific CD4 T cells (45). The anti-inflammatory activities of the Vi capsular polysaccharide and the TviA regulatory protein help explain why typhoid fever is characterized by a median incubation period of 2 weeks (46), suggesting that the initial dissemination of S. Typhi does not elicit innate host responses severe enough to produce symptoms of infection. In contrast, non-typhoidal Salmonella serovars, such as S. Typhimurium, which lack the Vi capsular polysaccharide and the TviA regulatory protein, have an incubation period of less than 24 h (47), illustrating that the initial invasion of the intestinal mucosa by these pathogens elicits a severe acute inflammatory response that swiftly manifests in symptoms of gastroenteritis.

## MATERIALS AND METHODS

### Bacterial strains and culture conditions.

Vi-positive (Vi^+^) S. Typhi isolate Ty2 (ATCC 19430) was obtained from the American Type Culture Collection. The Δ*tviB-vexE* mutant (strain SW74) has been previously described (Winter et al. [42]). A plasmid encoding GFP (pDW5) has been described previously (48) and was introduced into S. Typhi strains by electroporation.

Bacterial cultures were routinely incubated with aeration at 37°C in Luria-Bertani (LB) broth (10 g tryptone, 5 g yeast extract, 10 g Na/CL/L) or on LB agar plates unless indicated otherwise. Antibiotics were added as appropriate. To induce expression of the Vi capsular polysaccharide, low sodium (0.25% NaCl) was used to preculture bacterial strains. For all *in vitro* assays, S. Typhi strains were routinely prepared by inoculating LB broth with an overnight culture, followed by culturing for approximately 3 h until an optical density at 600 nm (OD_600_) of 1.0 was reached.

### Primary cells and tissue culture.

The University of California Davis Institutional Review Board approved the protocol for obtaining blood draws for this study, and informed consent was obtained from blood donors. Human neutrophils were isolated from the peripheral blood of healthy adult donors using an EasySep Direct Human Neutrophil isolation kit (STEMCELL Technologies, Inc.) in accordance with the manufacturer’s instructions. Neutrophils were cultured in RPMI 1640 medium supplemented with 10% heat-inactivated fetal bovine serum (FBS) and GlutaMAX Supplement (Gibco, no. 35050061), at 37°C in 5% CO_2_.

Human monocytes were isolated from the peripheral blood of healthy adult donors using an EasySep Direct Human Monocyte isolation kit (STEMCELL Technologies) or purchased frozen (STEMCELL Technologies). Following isolation of fresh human monocytes, the monocytes were differentiated into macrophages. Monocytes were cultured with 50 ng/mL human M-CSF (STEMCELL Technologies) in ImmunoCult-SF Macrophage Medium (STEMCELL Technologies) for 4 days to generate monocyte-derived macrophages.

THP-1 human monocyte-like cells were cultured in RPMI 1640 medium supplemented with 10% heat-inactivated FBS, GlutaMAX Supplement (Gibco, no. 35050061), 10 mM HEPES, and 1 mM sodium pyruvate at 37°C, 5% CO_2_. THP-1 cells were routinely differentiated by incubating in medium containing 20 ng/mL phorbol 12-myristate 13-acetate (PMA) for 3 days in an incubator (37°C, 5% CO_2_).

HeLa cells were cultured in RPMI 1640 medium supplemented with 10% FBS and GlutaMAX Supplement (Gibco, no. 35050061) at 37°C, 5% CO_2_. To express DC-SIGN in HeLa cells, an untagged DC-SIGN expression vector (HG10200-UT, Sino Biological) was transfected. Transfected cells were selected by treatment with 200 μg/mL Hygromycin B. To obtain single cells, the transfected cells were diluted to a concentration of 10 cells/mL (limiting dilution), and 100 μL of the cell suspension was seeded in each well of a 96-well plate. The presence of singlet cells in a well was confirmed by microscopy. DC-SIGN-expressing HeLa cell lines were evaluated and selected by quantitative real-time PCR (qPCR) assay.

### Gentamicin protection assays.

To determine bacterial uptake by phagocytes, primary human neutrophils, primary human monocyte-derived macrophages, or THP-1 human monocyte-like cells were infected with S. Typhi. Cells were seeded in 24-well plates at a density of 5 × 10^5^ cells per well. Bacteria were added to cells at a multiplicity of infection (MOI) of 10 bacteria per cell. The plate was centrifuged at 250 × *g* for 5 min and incubated (37°C, 5% CO_2_) for 30 min. After this incubation, RPMI medium (0.5 mL) containing 0.1 mg/mL gentamicin (Gibco) was then added to the cells for 30 min at 37°C to kill extracellular bacteria. Cells were washed three times with 0.5 mL of PBS and then lysed with 0.5 mL of pre-chilled sterile water for 15 min. The recovery of bacteria from phagocytes was quantified by spreading serial 10-fold dilutions onto LB agar plates with the appropriate antibiotics to enumerate CFU.

For blocking antibody assays, gentamicin protection assays were performed as described above with the addition of either 5 μg of anti-human DC-SIGN/CD209 antibody (clone no. 120507; R&D Systems) or 5 μg of anti-mouse IgG2B isotype control antibody (clone no. MAB004; R&D Systems) per well for 30 min prior to infection with bacterial strains.

### Bacterial binding assays.

To enumerate bacteria bound to cells, HeLa cells were infected with nonopsonized S. Typhi, and then cells were centrifuged at 250 × *g* for 5 min and incubated (37°C, 5% CO_2_) for 30 min to allow bacterial contact with the cell surface. After incubation, infected cells were vigorously washed five times with PBS to remove loosely associated bacteria on the cells. Cells were lysed by incubation with pre-chilled water for 15 min and serial 10-fold dilutions were spread on LB plates to enumerate CFU.

### Visualization of engulfed bacteria by phagocytes.

Phagocytes (differentiated human neutrophils, human monocytes, or differentiated THP-1 cells) were infected with S. Typhi expressing GFP (MOI =10), immediately centrifuged at 250 × *g* for 5 min, and incubated (37°C, 5% CO_2_) for 30 min to allow phagocytes to take up bacteria. Next, 100 μg/mL gentamicin was added for 30 min to kill extracellular bacteria. Cells were then fixed with 4% paraformaldehyde, DNA was counterstained with Hoechst 33342, and bacteria engulfed by phagocytes were visualized by fluorescence microscopy (Zeiss Axio Observer).

To distinguish between intracellular (engulfed) and extracellular bacteria, THP-1 cells were infected with S. Typhi wild type expressing GFP (MOI =40) and fixed as described above. Fixed cells were treated with 0.1% saponin for 30 min or remained untreated, and then bacteria were stained with anti-Salmonella O antigen factor 12 (O12) rabbit serum (BD Difco, no. 227791) and Alexa Fluor 594 conjugated goat anti-rabbit IgG (Invitrogen, no. A-11012). DNA was counterstained with Hoechst 33342, and images were obtained by fluorescence microscopy (EVOS FL Auto Imaging microscope; Invitrogen).

### Biotinylation of Vi capsular polysaccharide.

Purification of the Vi antigen has been described previously (2, 49). A previously described method for labeling Vi capsular polysaccharide with hydrazide-biotin (50) was modified as follows. First, 2 mg/mL of purified Vi capsule polysaccharide from S. Typhi strain Ty2 (2, 49) was incubated on ice with 1 mL sodium metha-periodate solution for 30 min. To remove excess periodate, the buffer was exchanged by dialysis in PBS or using a >3K Amicon column (Merck Millipore) against PBS. Then, 9 parts (1,800 μL) of oxidized and buffer-exchanged Vi capsule was incubated with 1 part (200 μL) of 50 mM hydrazide biotin solution (Thermo Fisher Scientific) for 2 h. To separate the biotinylated Vi and free biotin, the buffer was exchanged by dialysis in PBS or using the column. Biotinylated Vi was confirmed by a dot blot assay as described previously (2) using Vi antiserum (BD Biosciences) and horseradish peroxidase (HRP)-conjugated streptavidin.

### Visualization of Vi capsular polysaccharide binding to phagocytes.

To visualize binding of biotinylated Vi capsular polysaccharide in tissue culture, differentiated THP-1 cells were incubated (37°C, 5% CO_2_) with 150 μL biotin (5 mM, 1.8 mg/mL) or 150 μL biotin-Vi (mixed 1 mg/mL Vi + 1.8 mg/mL biotin) for 2 h. Cells were washed 4 times with PBS and fixed with 4% paraformaldehyde. Cells were then washed 3 times with wash buffer (0.05% Tween 20 in PBS). Biotin-Vi and biotin were stained by FITC (fluorescein isothiocyanate)-conjugated streptavidin, and DNA was counterstained with Hoechst stain 33342. Binding of biotin-Vi and biotin was visualized by fluorescence microscopy (Zeiss Axio Observer).

### CLR-hFc ELISAs.

A library of 15 CLR-hFc fusion proteins has been described previously (29, 30). CLR-hFc fusion proteins were purified from the supernatants of transfected CHO cells as described previously (29, 30) using HiTrap Protein G HP columns (GE Healthcare, Piscataway, NJ). For ELISAs with purified Vi capsular polysaccharide, streptavidin-coated microplates were used. For biotinylated Vi screening ELISAs, streptavidin-coated microplates were coated with 1 μg of biotin alone or biotinylated-Vi antigen overnight at 4°C on a shaker. Then, 200 ng of each respective CLR-hFc fusion protein in lectin-binding buffer (50 mM HEPES, 5 mM MgCl_2_, and 5 mM CaCl_2_) was added to the biotin or biotinylated-Vi antigen and incubated for 1 h at room temperature. Next, a 1:5,000-diluted HRP-conjugated goat anti-human IgG antibody (Jackson ImmunoResearch Labs, West Grove, PA) in dilution buffer (1% IgG-free bovine serum albumin [BSA], 0.05% Tween 20 in PBS) was added for 1 h at room temperature. At each step, plates were washed three times with wash buffer (0.05% Tween 20 in PBS). Finally, the substrate solution 3,3′,5,5′-Tetramethylbenzidine (TMB) (Thermo Fisher Scientific) was added to the samples, and the reaction was stopped with 2.0 M sulfuric acid. Data were collected using a microplate spectrophotometer (Thermo Fisher Scientific) at a wavelength of 450 nm. At least four independent experiments were performed, each with technical duplicates. For titration ELISAs, streptavidin-coated microplates were coated with increasing amounts of biotin or biotinylated-Vi antigen and ELISAs were performed as described above.

For ELISA-based binding assays of formalin-killed bacteria, bacteria were killed by treatment with 10% buffered formalin for 10 min at room temperature (RT). Next, 1 × 10^7^ formalin-killed bacteria were coated on high-binding 96-well microtiter ELISA plates (Sigma-Aldrich) at 4°C overnight on a shaker. Wells were then blocked with 1% IgG-free BSA (Sigma-Aldrich) in PBS for 1 h at RT, followed by the addition of CLR-hFc fusion proteins at 200 ng/mL in lectin-binding buffer for 2 h at RT. Binding was determined as described above.

For inhibition assays, CLR-hFc fusion proteins were incubated with 10 mM EDTA buffer instead of lectin-binding buffer.

### Visualization of CLR-hFc binding.

Bacterial binding to CLR-hFcs was visualized by staining bacteria with the indicated fusion proteins at 200 ng/mL in lectin-binding buffer for 2 h at 4°C, followed by incubation with an anti-human IgG (Fc)-PE or Alexa Fluor 488-labeled antibody (Jackson ImmunoResearch Labs) diluted 1:5,000. When indicated, bacteria were also stained with Salmonella Vi antiserum (Bio-Rad) at 1:5,000 and an anti-rabbit Alexa Fluor 594 at 1:5,000. When GFP-expressing bacteria were not used, bacteria were visualized with Hoechst 33342 staining (1:600). Stained samples were mounted onto slides using a Cytospin (Thermo Fisher Scientific) for 10 min at 22 × *g*. Samples were then mounted with coverslips using proLong antifade mountant (Invitrogen). Binding was visualized by fluorescence microscopy (Zeiss Axio Observer).

### Quantitative real-time PCR.

To detect expression of DC-SIGN (*CD209*) by human monocyte-derived macrophages, THP-1 cells, and HeLa cells, cells were differentiated as described above. RNA was extracted using TRI Reagent from 3 × 10^6^ cells (Molecular Research Center).

To test whether the Vi capsular polysaccharide induces anti-inflammatory responses, 3 × 10^6^ differentiated THP-1 cells were treated with purified Vi capsular polysaccharide for 21 h at the designated concentrations in RPMI 1640 medium supplemented with 10% heat-inactivated FBS, GlutaMAX Supplement (Gibco, no. 35050061), 25 mM HEPES, and 25 mM sodium pyruvate (37°C, 5% CO_2_). Cells were washed with PBS and RNA was extracted using TRI Reagent to analyze expression of the *Il10*, *Il6*, and *Il23A* genes. For stimulation of cells with lipopolysaccharide, cells were treated with 10 ng/mL LPS (O-9) from S. Typhimurium (Sigma-Aldrich) alone or with Vi capsular polysaccharide treatment for 21 h, washed with PBS, and then RNA was extracted using TRI Reagent.

For inhibition of RAF-1, differentiated THP-1 cells were pre-treated with 5 mM GW5074 (Sigma-Aldrich) for 2 h. Cells were then incubated with 10 μg purified Vi capsular polysaccharide for 21 h. RNA was extracted using TRI Reagent.

cDNA from each RNA sample was generated by reverse transcription-PCR (RT-PCR). Transcription levels were determined using a ViiA7 Real-Time PCR System (Applied Biosystems) using SYBR Green PCR Master Mix (Applied Biosystems). GAPDH (glyceraldehyde-3-phosphate dehydrogenase) was used for normalization. The threshold cycle (ΔΔ*CT*) method was used to calculate fold changes between groups.

### Fluorescence-activated cell sorting analysis.

To verify expression of DC-SIGN on the macrophage cell surface, THP-1 cells stimulated with 20 ng/mL PMA for 0 or 3 days or human monocytes stimulated with 50 ng/mL M-CSF for 0, 3, and 6 days were stained with a Zombie Aqua Fixable Viability kit (BioLegend) and APC-labeled anti-human DC-SIGN/CD209 antibody (R&D Systems, no. FAB161A-025). Flow cytometric analysis of the stained cells was performed using FACSVerse (BD Biosciences), and DC-SIGN expression levels in live single cells were analyzed using FlowJo (BD Biosciences).

### Statistical analysis.

Ratios (i.e., fold change and percentages) were converted logarithmically prior to statistical analysis. All data are expressed as the geometric mean and standard error of the mean. Analysis of variance (ANOVA) was used for multiple comparisons and Student’s *t* tests were used for pairwise comparison. *P* < 0.05 was considered significant.

### Data availability.

The authors certify that they will comply with ASM’s Data Policy. Data will be made publicly available upon publication and upon request for peer review.
