# Supplementary figures and images for: The Vi Capsular Polysaccharide of Salmonella Typhi Promotes Macrophage Phagocytosis by Binding the Human C-Type Lectin DC-SIGN
Source: mBio. 2022 Oct 26;13(6):e02733-22. doi: 10.1128/mbio.02733-22 (PMC9765441; doi:10.1128/mbio.02733-22)

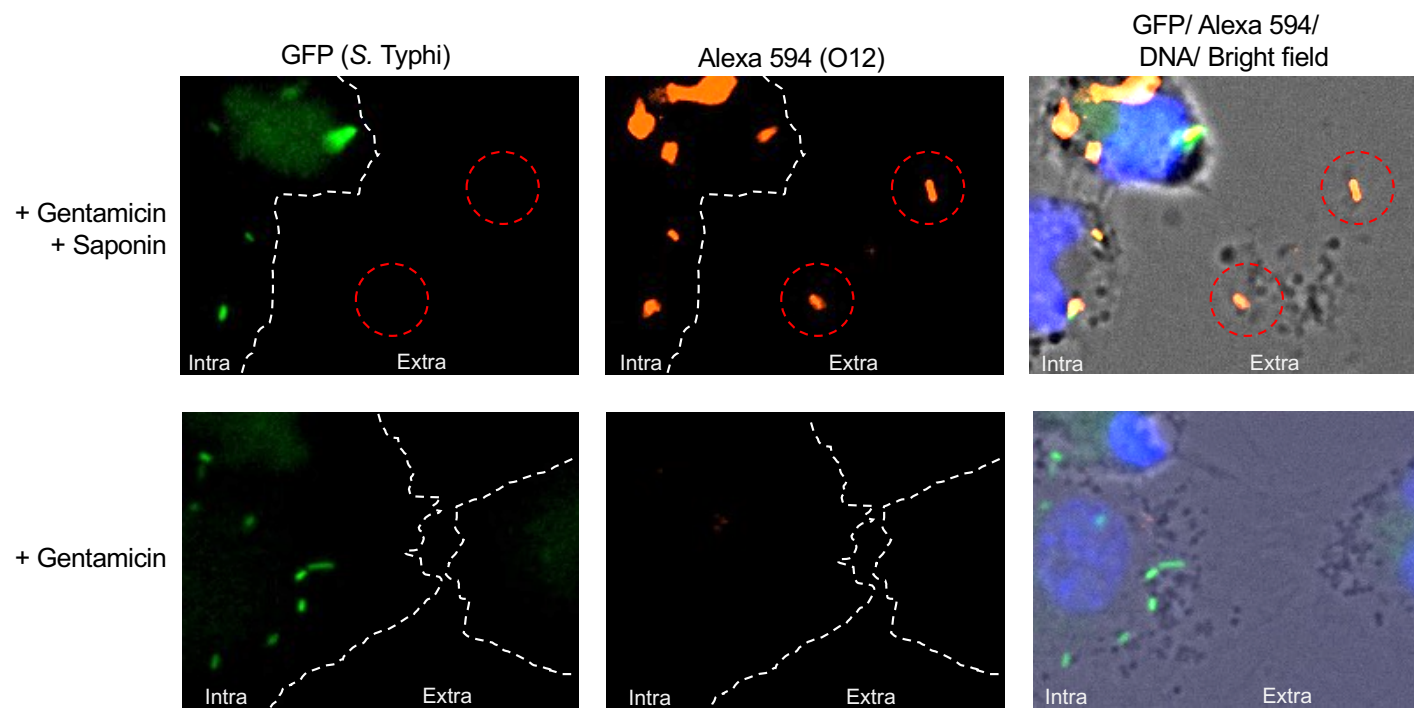

Supplementary figure S1

Supplement: FIG S1 [file mbio.02733-22-s0001.pdf]
